# Solid-state synthesis, magnetic and structural properties of interfacial B2-FeRh(001) layers in Rh/Fe(001) films

**DOI:** 10.1038/s41598-020-67837-2

**Published:** 2020-07-02

**Authors:** V. G. Myagkov, A. A. Ivanenko, L. E. Bykova, V. S. Zhigalov, M. N. Volochaev, D. A. Velikanov, A. A. Matsynin, G. N. Bondarenko

**Affiliations:** 1 Kirensky Institute of Physics, Siberian Branch of the Russian Academy of Sciences (SB RAS), 50/38 Akademgorodok, Krasnoyarsk, 660036 Russian Federation; 2 Institute of Chemistry and Chemical Technology, Siberian Branch of the Russian Academy of Sciences (SB RAS), 50/24 Akademgorodok, Krasnoyarsk, 660036 Russian Federation

**Keywords:** Magnetic materials, Magnetic properties and materials

## Abstract

Here we first report results of the start of the solid-state reaction at the Rh/Fe(001) interface and the structural and magnetic phase transformations in 52Rh/48Fe(001), 45Rh/55Fe(001), 68Rh/32Fe(001) bilayers from room temperature to 800 °C. For all bilayers the non-magnetic nanocrystalline phase with a B2 structure (nfm-B2) is the first phase that is formed on the Rh/Fe(001) interface near 100 °C. Above 300 °C, without changing the nanocrystalline B2 structure, the phase grows into the low-magnetization modification α_l_ʹ (M_S_^l^ ~ 825 emu/cm^3^) of the ferromagnetic α^ʹ^ phase which has a reversible α_l_ʹ ↔ αʺ transition. After annealing 52Rh/48Fe(001) bilayers above 600 °C the α_l_ʹ phase increases in grain size and either develops into α_h_ʹ with high magnetization (M_S_^h^ ~ 1,220 emu/cm^3^) or remains in the α_l_ʹ phase. In contrast to α_l_ʹ, the α_h_ʹ ↔ αʺ transition in the α_h_ʹ films is completely suppressed. When the annealing temperature of the 45Rh/55Fe(001) samples is increased from 450 to 800 °C the low-magnetization nanocrystalline α_l_ʹ films develop into high crystalline perfection epitaxial α_h_ʹ(001) layers, which have a high magnetization of ~ 1,275 emu/cm^3^. α_h_ʹ(001) films do not undergo a transition to an antiferromagnetic αʺ phase. In 68Rh/32Fe(001) samples above 500 °C non-magnetic epitaxial γ(001) layers grow on the Fe(001) interface as a result of the solid-state reaction between the epitaxial α_l_ʹ(001) and polycrystalline Rh films. Our results demonstrate not only the complex nature of chemical interactions at the low-temperature synthesis of the nfm-B2 and α_l_ʹ phases in Rh/Fe(001) bilayers, but also establish their continuous link with chemical mechanisms underlying reversible α_l_ʹ ↔ αʺ transitions.

## Introduction

An intriguing feature of the equilibrium diagram of the Fe-Rh system is the existence of the low-temperature transition at T_K_^αʺ→αʹ^ ~ 100 °C between antiferromagnetic (AFM) αʺ and ferromagnetic (FM) αʹ phases in a narrow (0.48 < x_Rh_ < 056) concentration interval in chemically ordered B2-FeRh alloys^1^. This transition is accompanied by an isotropic volume expansion of ~ 1% of the B2-FeRh unit cell, which not only radically changes the magnetic properties, but also changes the entropy^2,3^ and resistivity^4^. The magnetostructural αʺ → αʹ (AFM-FM) transition gives existence to the giant magnetostriction^5^ large magnetoresistance^6,7^ and magnetocaloric effects^8^ in B2-FeRh alloys. Numerous studies of bulk samples indicate that the starting temperature T_K_^αʺ→αʹ^ and characteristics of the αʺ → αʹ transition may be modified by variations the magnetic field^9,10^, microstructure^11^, thermal treatment^12^, energetic ion irradiation^13^, stress^14^ and hydrostatic pressure^15–17^ and can also be tuned over a wide temperature range (100–600 K) by chemical substitution^9,18^. Although bulk B2-FeRh samples exhibit a sharp transition with a small thermal hysteresis, thin films and nanoparticles often have an incomplete and broad asymmetrical hysteresis AFM-FM transition^1,19–34^. The starting transition temperature T_K_^αʺ→αʹ^ and magnetic properties in B2-FeRh films depend not only on compositional variations^26,27,34^, but are also highly sensitive to the type of substrate^19,24–26,29,35^, the thickness of the thin film samples^20,24,30^, the nature underlayers^33,36^ and the capping layers^31^. Significant stresses arise at the film/substrate interface^20,24^, which makes it possible to control the magnetic and electrical transport properties of B2-FeRh films on ferroelectric substrates^25,37–40^ by varying the external electric field. Many groups using magnetron sputtering or molecular beam epitaxy achieved epitaxial B2-FeRh(001) films grown on MgO(001) crystalline substrates. In most studies high-temperature heat treatments above 600 °C were used to obtain the high crystalline quality of chemically B2-ordered phase^32,33^. However, chemical interactions of Rh with Fe and solid-state synthesis of the Rh-Fe phases at the Rh/Fe interface remain completely uninvestigated.

In this work, we describe the solid-state reactions between polycrystalline Rh with epitaxial Fe(001) films in 52Rh/48Fe(001), 45Rh/55Fe(001) and 68Rh/32Fe(001) bilayers with 52Rh:48Fe, 45Rh:55Fe and 68Rh:32Fe atomic ratios, respectively. Under these conditions, the reaction products of 52Rh/48Fe(001) fall into the (0.48 < x_Rh_ < 056) concentration interval and the reaction products for 45Rh/55Fe(001) and 68Rh/32Fe(001) samples lie in Fe-rich and Rh-rich regions of the Fe–Rh system. The main purpose of this article is to show the complex and intricate nature of the B2-FeRh phases synthesis, which begins to form on the Rh/Fe interface at ~ 100 °C.

## Results

### Structural and magnetic phase transformations in 52Rh/48Fe(001) bilayer during annealing up 800 °C

**Figure 1:**
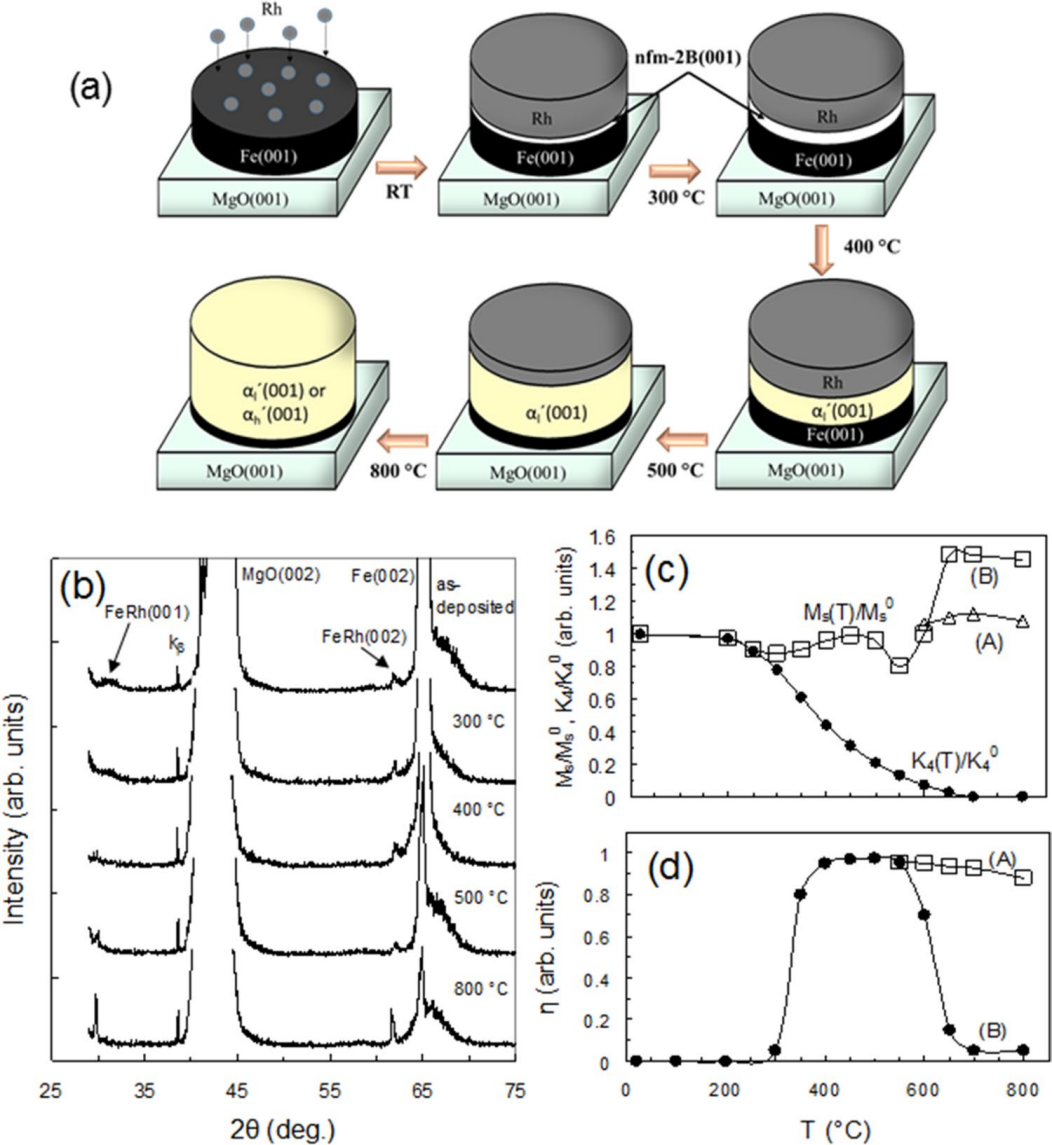
Low-temperature formation and evolution of B2-FeRh phases in the 52Rh/48Fe(001) bilayer. (**a**) Schematic of the phase transformations in the 52Rh/48Fe(001) bilayer showing the successive formation of the thin non-magnetic nanocrystalline epitaxial B2-FeRh (nfm-B2) film at ~ 100° C, which evolves into the low-magnetization modification α_l_ʹ of the ferromagnetic αʹ phase above 300 °C. After annealing above 600 °C the α_l_ʹ phase develops either into the α_h_ʹ phase with high magnetization or remains in the α_l_ʹ phase. (**b**) XRD patterns of B2-FeRh after annealing at different temperatures. (**c**) Evolution of the magnetization M_S_/M_S_^0^ and anisotropy K_4_(T_a_)/K_4_^0^ constants as a function of annealing temperature showing the low-temperature formation and mutual transformations of the B2-FeRh phases during the solid-state reaction between the Rh and Fe(001) layers. (**d**) Temperature dependence of the α_l_ʹ → αʺ transition degree η(T_a_) of B2-FeRh in the 52Rh/B2-FeRh/48Fe(001) trilayer confirming that B2-FeRh is a low-magnetization α_l_ʹ phase in the 300–600 °C temperature range.

Figure 1a shows a schematic diagram of the phase transformations consistently occurring in 52Rh/48Fe(001) bilayers on a MgO(001) substrate during annealing from room temperature to 800 °C which builds on the X-ray diffraction (XRD) analysis (Fig. 1b) and magnetic measurements (Fig. 1c,d). The transformations consist of the formation of a nonferromagnetic B2-FeRh phase (nfm-B2) thin layer at the Rh/Fe interface of the as-deposited sample, which above 300 °C turns into the α_l_ʹ phase. As will be shown below, the ferromagnetic αʹ phase may have two B2 modifications with similar or equal lattice parameters: a low-ferromagnetic α_l_ʹ having a magnetization ~ 825 emu/cm^3^ and a fully reversible α_l_ʹ ↔ αʺ (AFM-FM) transition and a high-ferromagnetic α_h_ʹ with a magnetization ~ 1,220 emu/cm^3^ and with a completely depressed α_h_ʹ → αʺ transition. As the annealing temperature increases above 600 °C the reaction products contain a mixture of epitaxial α_l_ʹ(001) and α_h_ʹ(001) grains. Figure 1b shows the XRD profiles of the as-deposited 52Rh/48Fe(001) film and after annealing at temperatures from room temperature to 800 °C. As-deposited samples show a strong Fe(002) peak, proving the epitaxial Fe(001) film growth on the MgO(001) substrate, and wide and very low diffraction peaks (001), (002) located at ~ 30° and ~ 62°, which are a signature of insignificant mixing, a reaction between the Rh and Fe(001) layers and the synthesis of a very thin epitaxial layer of nanocrystalline B2-ordered FeRh phase. These (001), (002) reflections were greatly broadened, split and their intensity did not change up to 600 °C. This strongly suggests that the reaction starts with the formation of (001)-textured nanograins containing B2—ordered FeRh phases with close lattice parameters and significant lattice distortions. After annealing above 600 °C the Fe(002) peak decreases significantly and practically disappears at 800 °C, which points to the complete termination of the reaction between the Fe and Rh layers. At the same time the (001), (002) peaks grow insignificantly, which is due to the annealing-induced coarsening of the B2—ordered FeRh nanograins. Using Scherrer’s formula the average grain size was estimated to be ~ 10 nm and slightly increased to ~ 40 nm with an increase in temperature from 600 to 800 °C. This means that the final product is a low crystalline quality epitaxial B2—ordered FeRh (001) layer on Mg(001). The asymmetric XRD scans demonstrate the orientation relationship of the B2-FeRh (001)[001] || Fe(001)[001]||MgO(001)[011] (see Supplementary Fig. 1). The order parameter was estimated S = 0.90 ± 0.02 for the synthesized B2-FeRh samples at temperatures in the 600–800 °C range. Figure 1c shows the dependence of the in-plane relative magnetic anisotropy constant K_4_(T_a_)/K_4_^0^ and the M_S_(T_a_)/M_S_^0^ relative magnetization as a function of the annealing temperature T_a_ {where for 52Rh/48Fe(001) samples K_4_^0^ = 2.4∙10^5^ erg cm^3^ and M_S_^0^ = 825 emu/cm^3^, see “Methods”}. With an increase in the annealing temperature T_a_ the constant K_4_(T_a_) monotonically decreases and becomes zero within experimental accuracy after annealing at 800 °C. This means that the first magnetic anisotropy constant K_1_ of the B2—ordered FeRh phase is zero or less than the measurement error (0.2 × 10^4^ erg/cm^3^). A value of K_1_ ~ 0 is consistent with the FeRh single films exhibiting an in-plane easy axis of magnetization with no measurable magnetocrystalline anisotropy^41^. Therefore, the K_4_(T_a_)/K_4_^0^ dependence can be used to find the thickness, magnetization and magnetic moment of the reacted Fe(001) layer after annealing at T_a_ (Supplementary Note 1). The dependence of the relative magnetization M_S_/M_S_^0^ as a function of annealing temperature T_a_ (Fig. 1c) has an unusual shape and convincingly shows complex and intricate scenarios of various phase formation and their mutual transformations in the Rh/Fe(001) bilayer during annealing to 800 °C. Within experimental error the relative anisotropy constant K_4_(T_a_)/K_4_^0^ and the relative magnetization M_S_(T_a_)/M_S_^0^ decrease identically as the annealing temperature increases up 300 °C. The synchronous decrease of the K_4_(T_a_)/K_4_^0^ and M_S_(T_a_)/M_S_^0^ values clearly proves the onset of the slow mixing of the Rh and Fe layers and the synthesis of the nonferromagnetic B2-FeRh(001) phase (nfm-B2) on the Rh/Fe(001) interface, whose volume increases with an increase in annealing temperature to 300 °C. Above 300 °C the magnetization increases to M_S_/M_S_^0^ ~ 1, which indicates a phase transformation of the nfm-B2 to the ferromagnetic α_l_ʹ phase. From Fig. 1c follows that the trilayer system Rh/α_l_ʹ/Fe (001) has the magnetization of the initial sample M_S_^0^ = 825 emu/cm^3^ (M_S_/M_S_^0^ ~ 1) in the temperature range (350–500 °C), which means that only the Fe atoms contribute to the saturation magnetization of α_l_ʹ-FeRh. Surprisingly, after annealing at 550 °C, the saturation magnetization decreases to 660 emu/cm^3^ (~ 20%), but the reaction product layer participates completely in the reversible α_l_ʹ ↔ αʺ transition (Fig. 1d). At annealing temperatures above 500 °C, the films begin to partially peel off from the substrate due to the strong stresses arising during the synthesis of the epitaxial α_l_ʹ-FeRh (001) film at the α_l_ʹ-FeRh(001)/Fe(001) interface. Therefore, a possible explanation is the deformation of some α_l_ʹ-grains with a loss of ferromagnetic order. However, the question about the exact origin of the decrease of M_S_/M_S_^0^ at 550 °C remains open. All of the 52Rh/48Fe (001) samples had the same temperature dependencies of the magnetization M_S_ (T_a_) up to 600 °C. However, after annealing above 600 °C the magnetizations of M_S_ sharply increased and assumed values that centered either around M_S_^1^ ~ 1,220 emu/cm^3^ (M_S_/M_S_^0^ ~ 1.47, B-samples) or M_S_^2^ ~ 940 emu/cm^3^ (M_S_/M_S_^0^ ~ 1.15, A-samples) (Fig. 1c).

### Temperature dependence of the reversible AFM-FM transition in the 52Rh/48Fe bilayer

In experiments, all 52Rh/48Fe(001) samples, after annealing each in a temperature range of 100–800 °C, were subjected to an investigation of the αʹ ↔ αʺ transition, consisting of measurements of the magnetic moment m(T_a_) of the synthesized layer B2-FeRh in the Rh/B2-FeRh/Fe (001) trilayer by the torque method at room temperature, after annealing at temperature T_a_ and after cooling in liquid nitrogen m^N^(T_a_). To characterize the reversible αʹ ↔ αʺ transition, we introduce the value η = 1 − m^N^(T_a_)/m(T_a_) for which η = 0 is absence of transition and η = 1 is perfect transition (see “Methods”). Figure 1d shows the temperature dependence of the degree η(T_a_) of the αʹ ↔ αʺ transition which has values (200–300 °C) η = 0, (400–600 °C) η > 0.95 and (700–800 °C) η = 0 for the (B) samples and η = 0.95–0.8 for the (A) samples in the specified temperature ranges. In the temperature interval 200–300 °C, the equality η = 0 is determined by the formation of a non-magnetic nfm-B2 phase. Although the α_l_ʹ (001) layer is located between the Rh and Fe (001) layers and grows epitaxial on the surface of Fe (001) in the (400–600 °C) interval it has a reversible α_l_ʹ ↔ αʺ transition with a low residual magnetization (see Supplementary Fig. . 2). In the (650–800 °C) interval the reaction ends and only the (A) samples reveal a reversible α_l_ʹ ↔ αʺ transition, while this transition does not occur in the (B) samples. We hypothesize that the αʹ phase has two B2-ordered polymorphic modifications having similar lattice parameters, but different magnetic properties. In the first modification the α_l_ʹ phase has a low magnetization about 825 emu/cm^3^, which is defined only by the Fe atoms with the Rh atoms not contributing to the magnetization, and the α_l_ʹ undergoes a complete reversible α_l_ʹ ↔ αʺ transition (Supplementary Fig. 3a). In contrast, in the second α_h_ʹ modification, which is primarily in the (B) samples, the Fe atoms supposedly polarize the Rh and the Rh atoms make the contribution to the saturation magnetization 1,220 emu/cm^3^ and in the α_h_ʹ phase the α_h_ʹ ↔ αʺ transition is completely suppressed (Supplementary Fig. 3b). Under such conditions, with an increase in temperature above 650–800 °C, some low-magnetic α_l_ʹ nanograins turn into high-magnetic α_h_ʹ and therefore, the samples have a magnetization between 825 and 1,220 emu/cm^3^ and exhibit an imperfect reversible transition with a residual magnetization. To summarize, the chemical reaction between Fe and Rh arises on the Fe/Rh interface at a low annealing temperature (~ 100 °C) and with an increase in annealing temperature the phase sequence 52Rh/48Fe → (~ 100 °C) nfm-B2 → (300 °C) α_l_ʹ → (600 °C) α_l_ʹ or α_h_ʹ is formed as shown in Fig. 1a.

### The phase and magnetic evolution in the 45Rh/55Fe(001) bilayer during annealing up 800 °C

**Figure 2:**
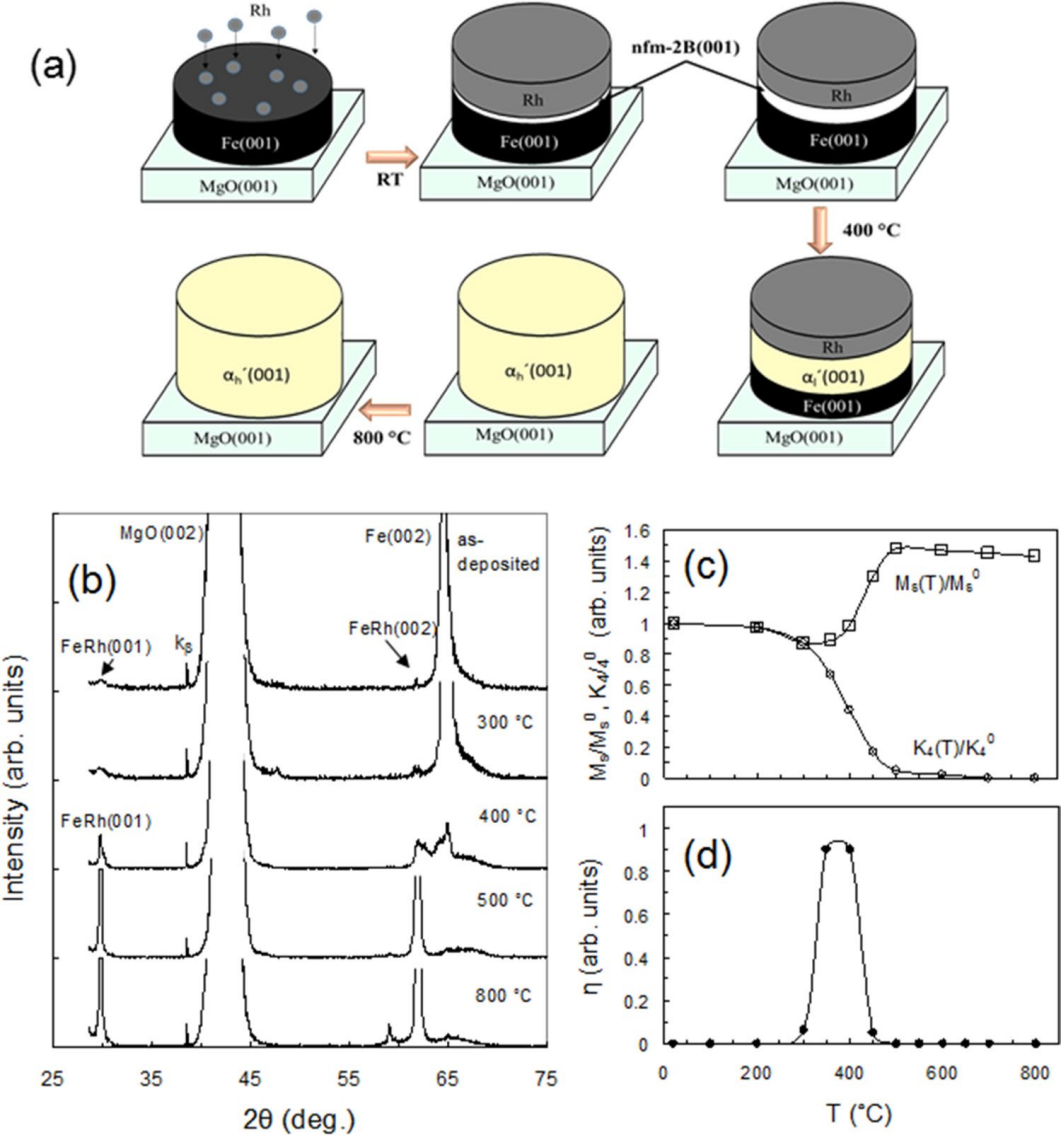
Evolution of the B2-FeRh phases in the 45Rh/55Fe(001) bilayer under annealing. (**a**) Schematic of the phase transformations in the 52Rh/48Fe(001) bilayer, also showing the same nfm-B2 and α^ʹ^ phase formation in 45Rh/55Fe(001) up to 450 °C. After annealing above 450 °C the α_l_ʹ develops into the α_h_ʹ phase as a result of the solid-state reaction α_l_ʹ + Fe → (~ 450 °C) α_h_ʹ. (**b**) Temperature-dependent XRD of the 45Rh/55Fe(001) bilayer from room temperature to 800 °C, indicating the phase transition from nanocrystalline α_l_ʹ to the highly crystalline α_h_ʹ phase above 450 °C. (**c**) Temperature-dependent magnetization M_S_/M_S_^0^ and anisotropy K_4_(T_a_)/K_4_^0^ constants confirming the sequential formation of the non-magnetic nfm-B2, low-magnetization α_l_ʹ and high-magnetization α_h_ʹ phases. (**d**) Temperature dependence of the α_l_ʹ → αʺ transition degree η(T_a_) indicating the existence of α_l_ʹ in the 300–450 °C temperature range.

The schematic diagram in Fig. 2a is based on the results presented in Fig. 2b–d and shows the same nonferromagnetic nfm-B2 phase which was formed in the 45Rh/55Fe and 52Rh/48Fe bilayers during annealing from room temperature to 300 °C. Above 300 °C the nfm-B2 phase consistently turns into ferromagnetic low-magnetization α_l_ʹ, which above 450 °C grows into the high-magnetization α_h_ʹ phases. As shown in Fig. 2b after annealing above 400 °C the (001) and (002) peaks start to grow and become very strong above 500 °C, which indicates the formation of high structural quality epitaxial α_h_ʹ(001) layers on the MgO(001) surface. The K_4_(T_a_)/K_4_^0^ and M_S_/M_S_^0^ dependencies (where for 45Rh/55Fe(001) samples K_4_^0^ = 2.5 × 10^5^ erg cm^3^ and M_S_^0^ = 875 emu/cm^3^, see “Methods”) presented in Fig. 2c show that the nanocrystalline phase α_l_^ʹ^(001) with low magnetization (M_S_/M_S_^0^ ~ 1.0) grows into the high quality epitaxial phase α_h_ʹ(001) with high magnetization (M_S_/M_S_^0^ ~ 1.47) above 450 °C. The high quality of the chemical ordering of the α_h_ʹ(001) films after annealing at 500 °C and 800 °C supports the order parameter S = 0.96 ± 0.02, which is more than the S = 0.90 ± 0.02 for α_l_ʹ in 52Rh/48Fe films. This result is unexpected, since the α_l_ʹ exists in a narrow composition range of nearly equiatomic concentration and must have a more complete B2 order than the Fe-rich α_h_ʹ phase. The α_h_ʹ(001) and α_l_ʹ(001) films have the same orientation relationship with the substrate MgO(001) (Supplementary Fig. 1). Above 500 °C the Fe has completely reacted with the Rh as evidenced by the K_4_(Ta)/K_4_^0^ dependence (Fig. 2c). As can be seen from Fig. 2d the degree of the AFM-FM transition η ~ 0.9, which means the α_l_^ʹ^ phase exists in a narrow temperature range (300–450 °C) and the AFM-FM transition has a relatively low residual magnetization (see Supplementary Fig. 4). Figure 2d shows the absence of the AFM-FM transition (η = 0), which indicates the formation of a α_h_ʹ phase from the α_l_ʹ phase after annealing above 450 °C (see also Supplementary Fig. 5). From these facts it transpires that the Fe-rich α_h_ʹ phase formed from the equiatomic α_l_ʹ compound above 450 °C by the solid-state reaction α_l_ʹ + Fe → (~ 450 °C) α_h_ʹ. Finally, we proved that even a slight Fe doping of α_l_ʹ causes a chemical reaction between Fe and α_l_ʹ and the start of the synthesis of the α_h_ʹ phase. This suggests that the high magnetization α_h_ʹ phase occurring in the 52Rh/48Fe(001) bilayer (B samples) also has more Fe content than α_l_ʹ and explains the compositional heterogeneity arising as a result of the nonequilibrium reaction processes. Thus, we show the phase evolution 45Rh/55Fe → (~ 100 °C) nfm-B2 → (300 °C) α_l_ʹ → (450 °C) α_h_ʹ is induced by the solid-state-reaction method and the final reaction product is a highly B2-ordered phase α_h_ʹ, which has a high magnetization of 1,270 emu/cm^3^ and in which the reversible α_h_ʹ ↔ αʺ transition is completely suppressed.

### Phase formation sequence, magnetic and structural development in the 68Rh/32Fe(001) bilayer during annealing up 800 °C

**Figure 3:**
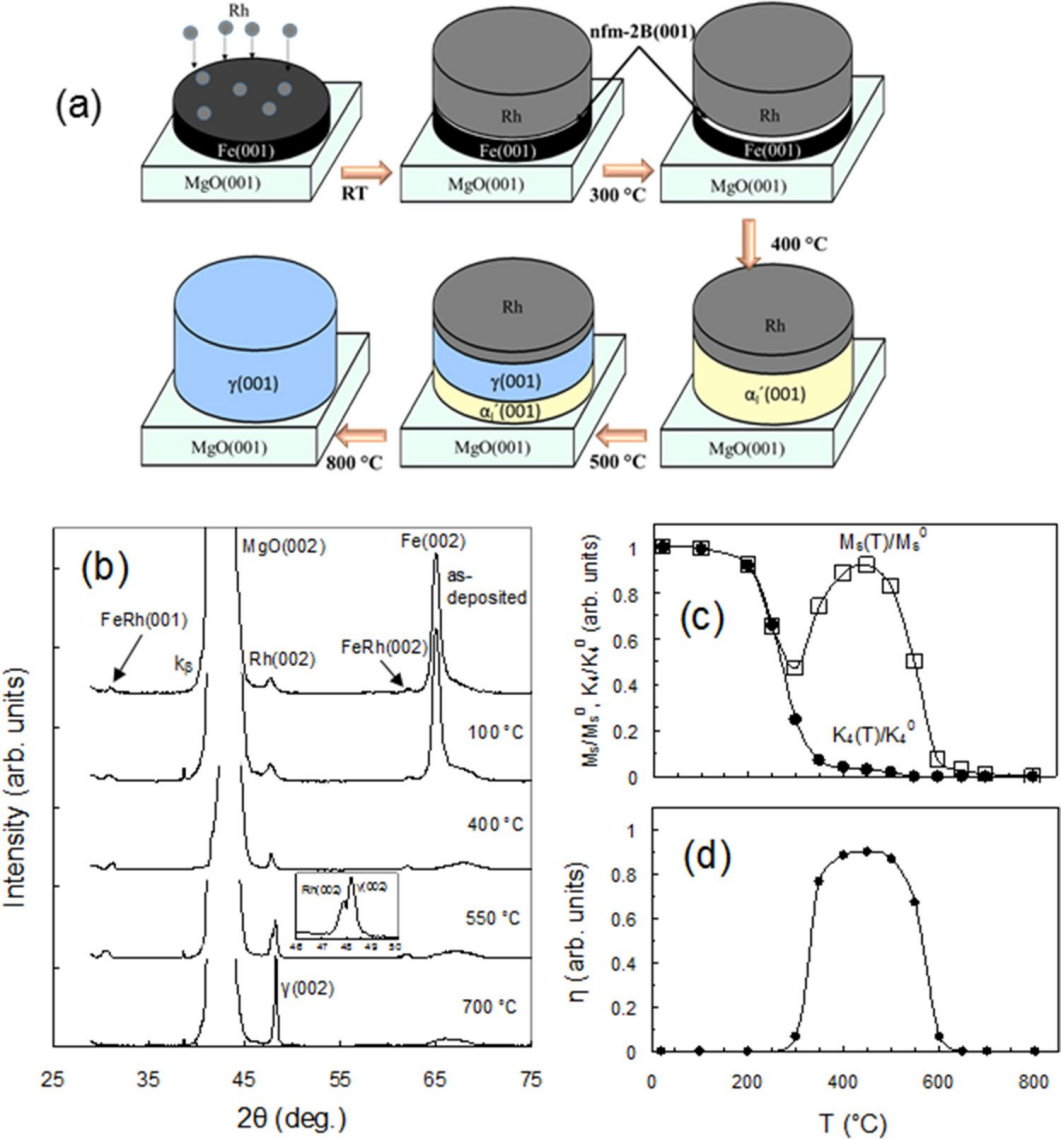
Phase evolution during solid-state reactions in the 68Rh/32Fe(001) bilayer. (**a**) Schematic of the phase transformations in the 68Rh/32Fe(001) bilayer as well as 45Rh/55Fe(001) and 52Rh/48Fe(001) bilayers, showing the same nfm-B2 and αʹ phase formation up to 550 °C. After annealing above 600 °C the epitaxial γ(001) layer begins to form on the Rh/α_l_ʹ(001) interface following the solid-state reaction Rh + α_l_ʹ → (550 °C) γ. (**b**) Temperature-dependent XRD indicating that only the epitaxial layer γ(001) is the final reaction product in the 68Rh/32Fe(001) bilayer. (**c**) Temperature-dependent magnetization M_S_/M_S_^0^ and anisotropy K_4_(T_a_)/K_4_^0^ constants confirming the sequential non-magnetic nfm-B2 and low-magnetization α_l_ʹ phase formation and the completion of the reaction between the Rh and Fe(001) layers after annealing at 500 °C. (**d**) Temperature dependence of the α_l_ʹ → αʺ transition degree η(T_a_) indicating the existence of α_l_ʹ in the 300–600 °C temperature range.

Figure 3a shows a schematic of the phase sequence formation in the 68Rh/32Fe(001) bilayer based on data presented in Fig. 3b–d. Figure 3a shows the same phase sequence including the sequential formation of the nanocrystalline nonferromagnetic nfm-B2 and the low-magnetization α_l_ʹ phases during annealing up to 500 °C in the 68Rh/32Fe, 52Rh/48Fe (Fig. 1a) and 55Rh/45Fe (Fig. 2a) bilayers. After annealing above 500 °C the epitaxial paramagnetic γ(001) phase starts to grow at the Rh/α_l_ʹ (001) interface (Fig. 3a). The XRD patterns show that after annealing at 400 °C the Fe layer forms an epitaxial α_l_′(001) layer in the Rh/α_l_ʹ(001) bilayer system (Fig. 3b, see Supplementary Fig. 6). Annealing above 500 °C the epitaxial γ(001) layer starts to grow on the α_l_′(001) surface originating from the solid-state reaction Rh + α_l_ʹ → (500 °C) γ. As shown in Fig. 3b the reaction is fully completed after 800 °C and the final reaction product is the γ(001) layer which has a cube-on-cube orientation relationship γ (001)[100]||MgO(001)[100] with the MgO(001) substrate. (Supplementary Fig. 7). The relative magnetization M_S_(T_a_)/M_S_^0^, and the K_4_(T_a_)/K_4_^0^ dependence presented in Fig. 3c confirm the sequential formation of nonferromagnetic nfm-B2 at about 100 °C, ferromagnetic α_l_ʹ above 300 °C and the paramagnetic γ phases above 500 °C. As follows from Fig. 3d, the ferromagnetic α_l_ʹ phase exists and undergoes a reversible AFM-FM transition (η ~ 0.9) in the 300–500 °C temperature range (Supplementary Fig. 6). Finally, we reveal the phase formation sequence *68*Rh/32Fe → (~ 100 °C) nfm-B2 → (300 °C) α_l_ʹ → (500 °C)γ during thermal annealing up to 800 °C and the final reaction product contains only an epitaxial γ(001) layer on the MgO(001) surface.

### Kinetic growth of the nanocrystalline nfm-B2 layer during thermal aging at 110 °C

**Figure 4:**
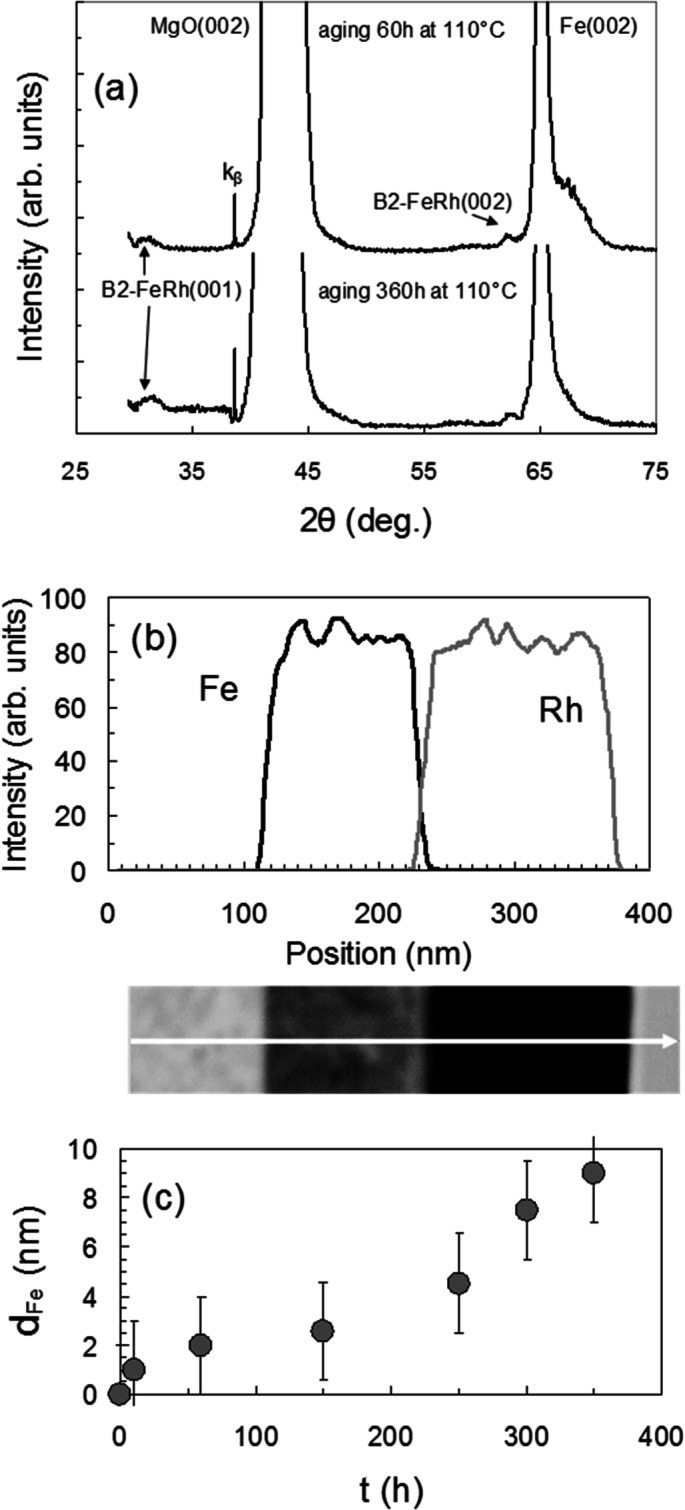
Direct evidence of the low-temperature synthesis of the thin interfacial nanocrystalline nfm-B2 layer in 52Rh/48Fe bilayers. (**a**) XRD pattern of the 52Rh/48Fe bilayer after aging at 110 ºC between 60 and 360 h. (**b**) TEM image of the cross-section and the EDS element mapping of the Fe and Rh in the 52Rh/48Fe bilayer after aging at 110 °C for 360 h. (**c**) The dependence of the reacted layer thickness h_Fe_ during aging up to 360 h at 110 °C.

To gain a better understanding of the origin and formation of the very thin nanocrystalline nfm-B2 layer at the Rh/Fe(001) interface at the temperature of the α_l_ʹ → αʺ transition, the synthesis kinetics of the nfm-B2 layer during isothermal aging at 110 °C was investigated. As stated above, deposition of Rh on Fe(001) at room temperature at any ratio of thicknesses between Rh and Fe leads to the formation of a thin interfacial nanocrystalline nfm-B2 layer. However, slight heating to 100 °C during sputtering cannot be excluded, therefore, aging of as-deposited samples was investigated at 110 °C. XRD data from the as-deposited Rh/Fe(001) bilayer and after aging up to 360 h shows a small broadened (001) superlattice and the fundamental (002) peaks of the nfm-B2 phase which did not change from aging time to 360 h (Fig. 4a). This means that long-time aging does not lead to the nfm-B2 grains coarsening and clearly demonstrates the inherent nanocrystalline nature of nfm-B2, which is also inherited by the α_l_ʹ phase. Figure 4b shows the TEM image and EDX line scan results of the Rh/Fe(001) bilayer aged at 110 °C for 360 h. The elemental profile obtained by the EDX line scan across the Rh/nfm-B2/Fe(001) trilayer shows an interfacial nfm-B2 thin film beside the initial Rh and Fe layers. The TEM image displays a compositional contrast that is proportional to the difference in the average atomic number of the respective areas. As a consequence, the reaction product nfm-B2 can be easily distinguished in the interfacial region from the initial Rh and Fe(001) areas. This allows one to estimate the thickness of the interfacial nfm-B2 h_nfm-B2_ ~ 15 nm (Fig. 4b). Figure 4c shows the thickness d_Fe_ of the Fe(001) layer, which entered into a reaction with Rh at 110 °C, as a function of aging time. There is a large experimental error in the thickness d_Fe_ measurements and therefore the dependence d_Fe_(t) is impossible to describe by an unambiguous kinetic equation, which suggests a possible growth mechanism. As shown in Fig. 4c the thickness d_Fe_ after aging for 360 h at 110 °C is ~ 8 nm, which corresponds to the thickness d_nfm-B2_ ~ 17 nm of the nfm-B2 layer (Supplementary Fig. 8a). This value agrees well with the estimated d_nfm-B2_ ~ 15 nm obtained from TEM‐EDX analysis. These findings are in good agreement with a previous report^42^ which showed and discussed the tendency towards intermixing and interfacial Rh–Fe alloy formation at the Rh/Fe interface at room temperature.

### Phase transition from nfm-B2 to α_l_′ at 300 °C

**Figure 5:**
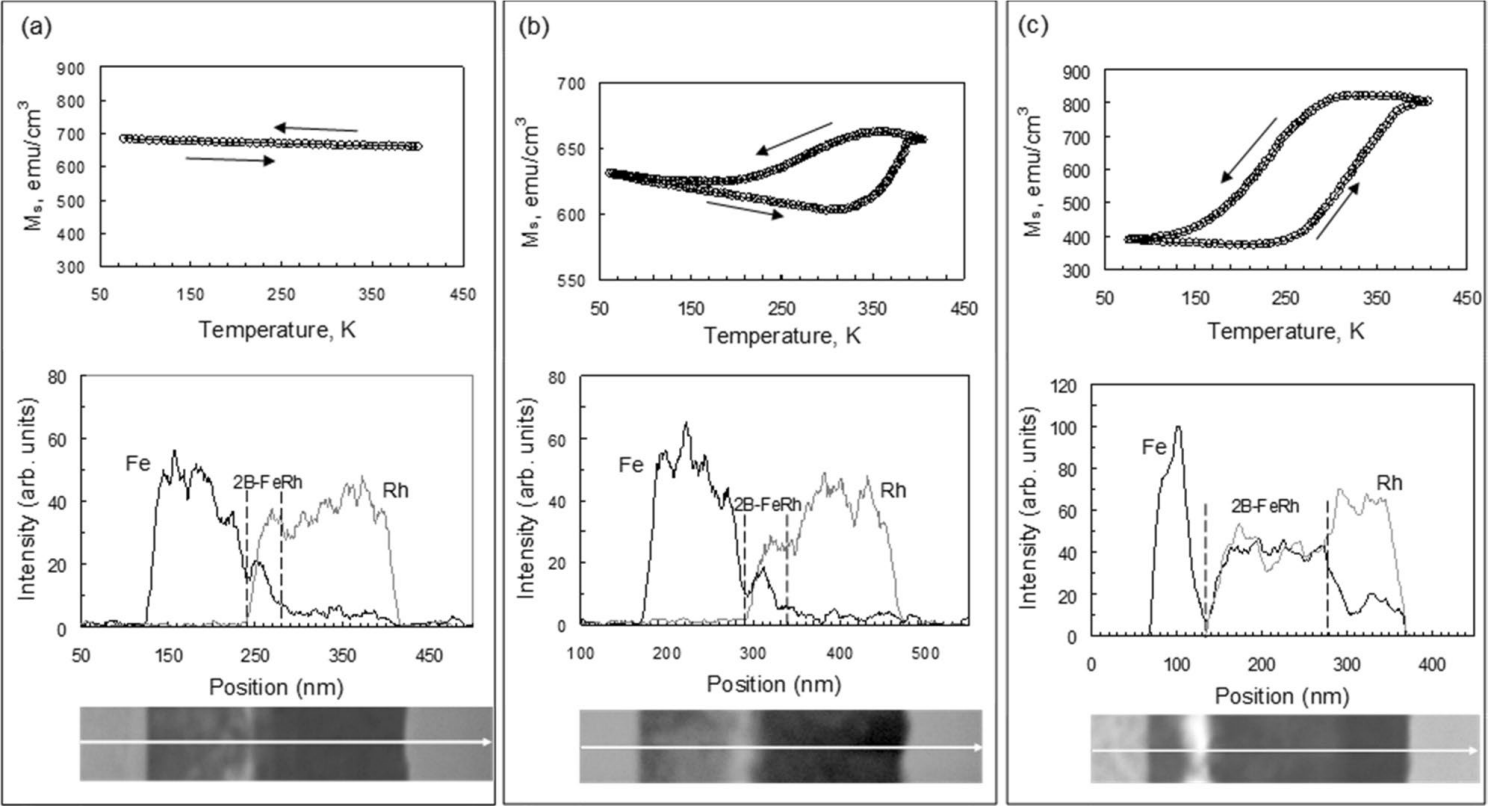
The nfm-B2 to α_l_′ phase transformation. TEM images of the cross-section, the EDS element mapping and the temperature dependent magnetization curves of the 52Rh/48Fe bilayer after aging (**a**) at 280 °C, (**b**) 300 °C, (**c**) 350 °C. The appearance of a loop in the M–T curve in (**b**) indicates the start of the ferromagnetic α_l_ʹ phase formation at 300 °C.

For a further understanding of the reactivity of Fe and Rh during the transition from nfm-B2 to α_l_ʹ around 300 °C, we investigated the evolution of the temperature dependencies of the magnetization and the thickness of the interfacial h_B2-FeRh_ layer in the 52Rh/B2-FeRh/48Fe(001) trilayer after annealing at 280 °C, 300 °C and 350 °C. Figure 5a shows the M–T curves for the 52Rh/nfm-B2/48Fe(001) trilayer after annealing at 280 °C which do not contain changes in either the forward or the reverse direction. This clearly demonstrates that the B2-FeRh interlayer remains a non-ferromagnetic nanocrystalline nfm-B2 layer after annealing in the 110–280 °C temperature interval. From the cross-sectional TEM image and the EDS line-scan (Fig. 5a), it can be seen that the d_nfm-B2_ interlayer in the 48Rh/nfm-B2/52Fe(001) trilayer increases to a thickness around d_nfm-B2_ ~ 30 nm after annealing at 280 °C. (Supplementary Fig. 8b). As shown in Fig. 5a,b slight increase in the annealing temperature to 300 °C leads to the appearance a road FM-AFM transition with a temperature hysteresis of magnetization about 80 K and a small increase in the d_B2-FeRh_ thickness of the B2-FeRh interlayer to ~ 40 nm (Supplementary Fig. 8c). The change in magnetization at the AFM-FM transition gives a rough estimate of the thickness d(α_l_ʹ) ~ 20 nm of the α_l_ʹ layer in the B2-FeRh interlayer. This clearly proves that after annealing at 300 °C the B2-FeRh interlayer contains a mixture of 50% nfm-B2 and 50% α_l_ʹ phases (Supplementary Note 2). Figure 5c shows that the thickness d_B2-FeRh_ grows quickly to 135 nm as the annealing temperature increases to 350 °C (Supplementary Fig. 8d). This value is in good agreement with the estimate obtained from the K_4_(T_a_)/K_4_^0^ dependence (Fig. 1c, see Supplementary Note 3). EDS analysis gives the average value of the atomic concentration ratio of Fe and Rh at about 1.0, which confirms that the reaction product layer is the B2-FeRh phase. Pores are visible on the Fe/nfm-B2 interface (Fig. 5c, Supplementary Fig. 8d), which are well known as Kirkendall voids. The explanation of the Kirkendall effect is based on the difference in rates of the reacting atoms and it proves that Fe is the dominant diffusing species during B2-FeRh formation. Figure 5c shows the M–T curve having a road FM-AFM transition with large thermal hysteresis width about 100 K, which often occurs in thin film system. This may be due to inhomogeneities of composition, stresses and nanocrystalline growth as a result of non-equilibrium synthesis of the B2-FeRh phases. Thus, this is proof that the reactivity of Fe and Rh is very low at 110 °C and begins to increase greatly during the transition nfm-B2 → α_l_ʹ above 300 °C. This causes strong temperature dependencies up to 500 °C of the relative magnetic anisotropy constant K_4_ (T_a_)/K_4_^0^ (Figs. 1, 2, 3) and electrical resistance (Supplementary Fig. 9).

## Discussion

Numerous experiments have proved that as temperature rises, only one phase appears on the interface of bilayer film systems, which is called the first phase^43–45^. The initiation temperatures T_in_ of the first phase for most bilayers lie below 400 °C. However, many thin-film reactions are initiated near room temperature and even occur at cryogenic temperatures. At such low temperatures diffusion is extremely small and cannot provide atomic transfer in a solid state^46–48^. This suggests an alternative view, in which not diffusion, but chemical interactions play a crucial role in the initiation and kinetics of solid state interfacial reactions. Under the influence of chemical interactions that arise above the initiation temperature T_in_, the chemical bonds break in the reactants, and the reacting atoms migrate into the reaction zone to synthesize new compounds. Previously, it was shown that the initiation temperatures T_in_ are close to or coincide with the solid-state transformation temperatures T_k_ of several reagent-based binary systems, such as order–disorder transitions, the superionic transition, the spinodal decomposition, martensitic transformations and others^46–48^. This suggests that the same chemical interactions underlie and control both the solid-state thin-film reactions and the corresponding solid-state transformations. The equality T_in_ = T_k_ indicates that low-temperature solid-state thin-film reactions in A/B bilayers occur only in A–B binary systems, which have corresponding low-temperature solid-state transformations. Therefore, the study of reactions in A/B bilayers with different layer ratios is a study of the low-temperature part of the A–B phase diagram.

It is well established that ordered B2 alloys, such as NiTi, AuCd, NiAl have reversible low-temperature martensitic transformations, in which the high-temperature austenite B2-phase develops into a low-temperature martensitic phase through a complex process of the* formation *of *intermediate phases.* We have shown earlier the initiation temperatures T_in_(Ti/Ni) < 150 °C^49^, T_in_(Ni/Al) ~ 180 °C^50^, T_in_(Cd/Au) = 67 °C^51^ in Ti/Ni, Ni/Al, Cd/Au bilayers, respectively. These temperatures are close to or coincide with the reverse martensitic transformation starting temperatures A_s_(B2-TiNi) ~ 100 °C, A_s_(B2-NiAl) ~ 180 °C, A_s_(B2-CdAu) = 67 °C. The reversible α_l_ʹ ↔ αʺ transition in B2-FeRh possesses all the characteristics of a martensitic transformation (α_l_′-austenite, αʺ-martensite), because it can pass at high speeds^52,53^, has isotropic volume changes at the transition^1^, can be induced by the application of stress^19,20,24,25,37–40^ and magnetic field^1,9,10^, has martensitic instabilities^36,54^ and the α_l_ʹ and αʺ lattices have a cube-on-cube orientation relationship. According to the phase diagram, the transition α_l_ʹ ↔ αʺ has a minimum temperature T_k_ ~ 100 °C among other structural transformations in the Fe-Rh system. From the above, we have concluded that the initiation temperature of the reaction T_in_(Rh/Fe) in the Rh/Fe bilayer coincides with the martensitic-like transition temperature T_K_ ~ 100 °C in B2-FeRh. The coincidence of the starting temperature of the reaction between Fe and Rh and the temperature of the magnetostructural AFM-FM transition suggests common chemical mechanisms behind both phenomena*,* but that connection remains to be confirmed by additional experimentation.

As mentioned above, the formation of compounds at the Rh/Fe interface starts in the temperature range 100–300 °C from the synthesis of the non-ferromagnetic nfm-B2 phase. Since this phase has only (001) and (002) B2-FeRh reflections, this means that nfm-B2 is either a martensitic-like antiferromagnetic αʺ-phase or a non-ferromagnetic martensitic variant which is stabilized by strains resulting from the non-equilibrium synthesis of the nfm-B2 phase. B2-FeRh, similar to other B2 phases of NiTi, AuCd, NiAl alloys, experiences premartensitic instabilities with the subsequent formation of the different structural variants of martensite. Our hypothesis is the amorphous phase appears above 100 °C at the Rh/Fe interface, which is then transformed into a nanocrystalline state containing B2 nanograins of martensite variants with lattice parameters close to αʺ martensite. This is consistent with the possible existence of various structural phases of FeRh, predicted by ab initio calculations^54,55^ and found in the experimental study^36^. Competition between the α_l_ʹ and αʺ phases during the partial crystallization suppresses grain growth and stabilizes the nano-grained B2 structures in an amorphous matrix. The formation of an amorphous phase is a quite common phenomenon in the initial stage of solid-state reactions in bilayers and multilayers, although the nature of this phenomenon is still a subject of dispute^57,58^. It is interesting to note the general features of the initial stage of the synthesis of B2 phases in Ti/Ni and Rh/Fe thin films. In Ti/Ni multilayers the amorphous phase starts near the martensitic transition temperature (~ 100 °C), which turns into B2-NiTi^59,60^ at annealing temperatures above 350 °C. Similar to Ti/Ni, the reaction in the Rh/Fe(001) bilayer starts at ~ 100 °C with the formation of the interfacial amorphous phase, which partially crystallizes. From this point the as-deposited films consist of nanocrystalline B2 grains dispersed in the amorphous matrix. This strongly suggests that both amorphous phases are amorphous martensite, which may be a universal phenomena of the solid-state synthesis of martensitic phases^61^. This scenario is different from the synthesis of B2-NiAl and B2-AuCd, which begin to form in Al/Ni^50^ and Cd/Au^51^ bilayers at martensitic transformation temperatures without the formation of an intermediate amorphous martensite.

Analysis of the general thermodynamic characteristics and features of B2-FeRh and B2-NiTi suggests the possibility of a fabrication of the B2-FeRh compound by self-propagating synthesis (Supplementary Note 3). Our approach assumes ~ 100 °C is the starting temperature of the formation of the B2 phase, which is associated with the reversible AFM-FM transition, and the synthesis temperatures T_in_(α_h_ʹ) =  ~ 450 °C and T_in_(γ) =  ~ 500 °C of the α_h_ʹ and γ phases coincide with the phase transition temperatures in the Fe-rich and Rh-rich regions of the Fe–Rh system, respectively. Such an approach is justified by us for the well-studied Fe–Ni system^62^ and made it possible to predict phase transformations in other binary metallic systems^46–48^. Therefore, further study of solid-state reactions in Rh/Fe films, depending on the composition, will make it possible to specify the low-temperature part of the Fe–Rh phase diagram, which still remains unknown^63^.

In conclusion, we have uncovered that regardless of the Rh and Fe thicknesses the thin nonmagnetic nanocrystalline B2-FeRh layer starts to form at ~ 100 °C and grows up to 300 °C on the Rh/Fe interface. Above 300 °C the nonmagnetic phase is converted into a low-ferromagnetic B2 α_l_ʹ modification of the α^ʹ^ phase with a magnetization ~ 825 emu/cm^3^ without changing the nanocrystalline structure. Above 500 °C the α_l_ʹ reacted with Fe and formed B2 α_h_ʹ with a magnetization ~ 1,270 emu/cm^3^ in the 45Rh/55Fe(001) samples and the α_l_ʹ reacted with Ph and formed the non-ferromagnetic γ phase in the 68Rh/32Fe(001)samples. Magnetic analysis has revealed that only the α_l_ʹ undergoes the complete reversible α_l_ʹ ↔ αʺ transition and there’s no transition in the α_h_ʹ samples. Thus, our work not only provides an idea of how phase sequences start and develop depending on the composition of the Fe/Rh bilayers, but also suggests the interesting possibility that a similar chemical mechanism may be at play behind the low-temperature reaction of Fe and Rh and the AFM-FM transition in B2-FeRh.

## Methods

### 52Rh/48Fe(001), 45Rh/55Fe(001) and 68Rh/32Fe(001) bilayers preparation and characterization

At first the epitaxial Fe(001)/MgO(001) films were grown on single-crystal MgO(001) substrates by a thermal evaporation method in a vacuum chamber at a pressure of 10–6 mbar. To obtain high-quality Fe(001) films, the substrates was previously outgassed at 300 °C for 1 h and the Fe layers were deposited at 250 °C. Epitaxial Fe (001) films had the orientation ratio Fe(001),[100]||MgO(001),[110] with the MgO(001) substrate and the magnetocrystalline anisotropy constant K_4_, which coincided with the value of bulk iron K_1_ = 4.9 × 105 erg/cm^3^. The constant K_4_ in the (001) plane was determined by a torque magnetometer in a magnetic field H = 12 kOe. The torque curve L_ǀǀ_(φ) in the (001) plane was determined according to the equation 2L_ǀǀ_(φ) = K_4_Vsin4φ + 2 K_u_ VSin(2φ + γ), in which, in addition to the dominant 4φ-term, there is a minor 2φ-term K_u_ term due to the surface roughness of the MgO(001) substrate and a slight misorientation of the Fe(001) grains. In the equation K_u_ is uniaxial anisotropy constant, V is the volume of the film, φ is the angle between the easy axis of fourfold anisotropy and the magnetization MS, γ is the angle between the easy axis of fourfold anisotropy and the axis of the uniaxial anisotropy. The K_4_V value was calculated from the torque curve L_ǀǀ_(φ) at the maximum of the 4φ-term: 2L_max_ = K_4_V. High quality epitaxial Fe(001)/MgO(001) films also can be obtained by various other methods as reported in the literature.

The starting Rh/Fe(001) bilayers were obtained by the evaporation of the Rh layers on Fe(001)/MgO(001) samples using dc sputtering in a magnetron sputtering system. The base pressure of the chamber was less than ~ 1 × 10^–6^ mbar, and a working pressure of ~ 1 mTorr Ar was used during sputtering. To prevent a reaction between Rh and Fe, the Rh layer was deposited at room temperature. Under such deposition conditions the polycrystalline Rh layer was formed on the Fe(001) surface. Three different types of samples were prepared for the experiments, namely Rh/Fe (001) bilayers with Rh-rich, approximately 1Fe:1Rh and Fe-rich atomic ratios, each with a total thickness about 300 nm. The elemental chemical composition determined by energy dispersion X-ray (EDX) analysis showed sample compositions of Rh_68_Fe_32_, Rh_52_Fe_48_ and Rh_45_Fe_55_, respectively. The saturation magnetization M_S_^0^ and the magnetic fourfold anisotropy constants K_4_^0^ were determined for the total volume of the 52Rh/48Fe, 45Rh/55Fe and 68Rh/32Fe bilayers, which turned out to be M_S_^0^ = 825 emu/cm^3^, K_4_^0^ = 2.2 × 10^5^ erg/cm^3^ for the 52Rh/48Fe bilayers, M_S_^0^ = 875 emu/cm^3^, K_4_^0^ = 2.4 × 10^5^ erg/cm^3^ for the 45Rh/55Fe bilayers and M_S_^0^ = 450 emu/cm^3^, K_4_^0^ = 1.25 × 10^5^ erg/cm^3^ for the 68Rh/32Fe bilayers.

The reversible AFM-FM phase transitions were checked using a superconducting quantum interference device (SQUID) magnetometer. Magnetic fields of H = 1.0 kOe were applied along the in-plane [100] MgO direction, which coincides with the easy axis of the Rh/Fe (001) bilayers, at all measurements in the 77–400 K temperature interval. The saturation magnetization M_S_ and the coercivity H_C_ were measured with a vibration magnetometer in magnetic fields up to 22 kOe. All saturation magnetization measurements were monitored using the torque method^64^.

The formed phases were identified with a DRON-4-07 diffractometer (CuKa radiation). The epitaxial relationships between MgO(001) and the B2, γ layers that formed in the reaction products were X-ray studied with a PANalytikal X'Pert PRO diffractometer with a PIXctl detector. CuKa radiation monochromatized by a secondary graphite monochromator was used in the instrument. The order parameter for the synthesized samples at temperatures in the 600–800 °C range was estimated using the equation S = (I_001_/I_002_)^1/2^/1.07, where I_001_ and I_002_ are experimental integrated intensities of superstructural (001) and fundamental (002) reflections^65^.

The cross-sectional samples for TEM studies were prepared by a focused ion beam (single-beam FIB, Hitachi FB2100) at 40 kV. In order to protect the surface of interest from milling by the Ga + ion beam during sample preparation, a Ge layer was deposited onto the Fe-Rh film before cross-sectional sample preparation by FIB. TEM studies were carried out using a Hitachi HT7700 TEM (acceleration voltage 100 kV, W source) equipped with a STEM system and a Bruker Nano XFlash 6T/60 energy dispersive X-ray (EDX) spectrometer. The imaging and EDX spectroscopy line scans and mapping were carried out in STEM mode with an electron probe of diameter ~ 30 nm.

### Characterization of solid-state phase transformations

The starting 52Rh/48Fe, 45Rh/55Fe and 68Rh/32Fe bilayers were annealed at temperatures ranging from 50 to 800 °C in increments of 50 °C. The samples were held at each temperature at a pressure of 10^−6^ Torr for 1 h. To characterize the phase transformations the crystal structure, the magnetic moments m^0^(T_a_) (where m^0^(300 K) = M_S_^0^V), the magnetic fourfold anisotropy constants K_4_(T_a_) and the degree of the αʹ → αʺ transition η(T_a_) were determined for all bilayers after annealing at each temperature T_a_. In order to fully characterize the phase transformations, cross-sectional SEM images and an elemental analysis of the phases of several samples using EDX were carried out.

### Measurement of the α_l_′ → α″ transition degree

In experiments, the degree η(T_a_) of the FM-AFM phase transition was determined for all 52Rh/48Fe, 45Rh/55Fe and68Rh/32Fe samples after annealing at each temperature in the range of 100–800 °C . The magnetic moments m^0^(T_a_) were measured by the torque method^64^, after annealing at temperature T_a_ and cooling in liquid nitrogen m^N^(T_a_), to find the value of the degree η(T_a_). The magnetic moment m(T_a_) of the synthesized α_l_ʹ—FeRh layer in the Rh/α_l_ʹ—FeRh/Fe(001) trilayer after annealing at temperature T_a_ is equal to the difference m(T_a_) = m^0^(T_a_)—K_4_(T_a_)m^0^/K_4_^0^ of the magnetic moments of the m^0^(T_a_) film and the K_4_(T_a_)m^0^/K_4_^0^ unreacted layer of the Fe(001) layer, where K_4_(T_a_)m^0^/K_4_^0^ is the magnetic moments of the unreacted Fe(001) layer. After placing the sample in liquid nitrogen, only the ferromagnetic α_l_ʹ—FeRh phase in the Rh/α_l_ʹ—FeRh/Fe(001) trilayer is subjected to the transition into the antiferromagnetic αʺ phase and the magnetic moment m(T_a_) is reduced to m^N^(T_a_). The quantity η = 1 − m^N^(T_a_)/m(T_a_) is a quantitative characteristic of the degree of the α_l_ʹ → αʺ transition, where η = 0 and η = 1 mean the absence of and the complete FM → AFM transition (residual magnetization is zero), respectively.

## Supplementary information

Supplementary file1 (DOCX 4798 kb)
