# Supplementary material for: Solid-state synthesis, magnetic and structural properties of interfacial B2-FeRh(001) layers in Rh/Fe(001) films
Source: Sci Rep. 2020 Jul 2;10:10807. doi: 10.1038/s41598-020-67837-2 (PMC7331715; doi:10.1038/s41598-020-67837-2)
Supplement: Supplementary file 1 — Supplementary file1 (DOCX 4798 kb) [file 41598_2020_67837_MOESM1_ESM.docx]

**Supplementary Materials**

**Solid-state synthesis, magnetic and structural properties of interfacial B2-FeRh(001) layers in Rh/Fe(001) films**

V.G. Myagkov, ^1,*^ A.A Ivanenko ^1^, L.E. Bykova ^1^, V.S. Zhigalov ^1^, M.N. Volochaev ^1^, D.A. Velikanov ^1^, A.A. Matsynin ^1^, G.N. Bondarenko ^2^

^1^ Kirensky Institute of Physics, Federal Research Center KSC SB RAS, Krasnoyarsk, Russia

^2^ Institute of Chemistry and Chemical Technology, Federal Research Center KSC SB RAS, Krasnoyarsk, Russia

**Supplementary Figure 1. XRD scan of an α_՜l_ (001) film on MgO(001). a** – XRD scan for the low-magnetization modification α_l_^ʹ^ (M_S_^1^ ~ 820 emu/cm^3^) (A-sample) of the epitaxial ferromagnetic α^ʹ^ (001) film, which was grown epitaxially on a MgO(001) substrate in a 45Rh/55Fe(001)/MgO(001) sample after annealing from 300° C to 800 °C. **b** - The φ-2θ asymmetric diffraction images for the α_l_^ʹ^ film and the MgO substrate. These scans reveal the α_l_^ʹ^ (001)[100]||MgO(001)[110] epitaxial orientation relationship. **c-**Schematic drawing illustrating the epitaxial relationship between the α_l_^ʹ^ and other B2-FeRh(001) films growing on the MgO(001) substrate.

**Supplementary Figure 2. Magnetization vs temperature curves extracted for the 52Rh/α_l_**^ʹ^**/48Fe(001) trilayer after annealing at 450 °C. a** –Temperature dependent magnetization curves of the Rh/α_l_^ʹ^/Fe (001) films showing the hysteresis in the AFM ↔ FM transition. Since the magnetization of **the 52Rh/48F (001) bilayer** is M_S_^0^ = 825 emu/cm^3^, using expression (3) from Supplementary Note 1 the magnetization ΔM(Fe) of the unreacted Fe(001) layer is ΔM(Fe) = 0.3 M_S_^0^ ~ 250 emu/cm^3^. Subtracting this value from the M-T curve for the Rh/α_l_^ʹ^/Fe (001) trilayer, creates the M-T curve of the α_l_^ʹ^ layer with residual magnetization ΔM. The relationship (ΔM(α_l_^ʹ^)-ΔM)/ΔM(α_l_^ʹ^) = 0.93 is the degree η of the α^ʹ^_l_ → α^ʺ^ transition which agrees well with the value η = 0.95 obtained by the torque method (**Fig. 1d** of the manuscript). The inset shows the in-plane magnetization M_S_ along the [100] direction (hard axis) as a function of the magnetic field H after cooling to nitrogen temperature and heating up to room temperature (300 K). In this instance the magnetization M_S_ corresponds to point A on the M-T curve. It is important to note that under such conditions the α_l_^ʹ^ layer in the Rh/α_l_^ʹ^/Fe (001) trilayer undergoes a transition into the antiferromagnetic α^ʺ^ layer, which is epitaxially and exchange coupled with the Fe (001) layer. However, the M-H curve did not contain an exchange bias field. Possible causes for the appearance and absence of the exchange bias field at the FeRh/FM interfaces are discussed in the papers [1,2].

**b** – TEM image of the cross-section and compositional profiles along the scan line shown in the TEM image of the cross-section of the Rh/α_l_^ʹ^/Fe (001) trilayer after annealing at 450 °C. Using expressions (2) and (6) from **Supplementary Note 1**, we can calculate the thicknesses of the reacted Fe (001) layer d_Fe_ ~ 90 nm and the d(α_l_^ʹ^) layer ~ 190 nm which form in the 52Rh/α_l_^ʹ^/48Fe (001) trilayer after annealing at 450 °C. The calculated value d(α_l_^ʹ^) ~ 190 nm agrees well with the experimental value obtained from the compositional profile presented in (b).

**Supplementary Figure 3. Magnetization vs temperature curves for A and B samples obtained by annealing 52Rh/48Fe(001) trilayers at 700 °C. a** – M-T curve for α_l_^ʹ^ films (A-sample, M_S_^1^ ~ 825 emu/cm^3^), showing the hysteresis in the AFM ↔ FM transition. **b -** In contrast to α_l_^ʹ^ films the M-T curve for α_h_^ʹ^ films (B-sample, M_S_^2^ ~ 1220 emu/cm^3^) has a high magnetization and does not show the AFM ↔ FM transition. The most unexpected result is the formation of the α_l_^ʹ^ and α_h_^ʹ^ phases having close lattice parameters obtained under the same conditions in the (0.48 < x_Rh_ < 0.56) concentration range. Therefore, the magnetic characteristics of the M-T curves depend on the ratio of the α_l_^ʹ^ and α_h_^ʹ^ phases and the residual magnetization is determined by the fraction of the α_h_^ʹ^ phase. Moreover, the magnetization of the B2-FeRh samples in the (0.48 < x_Rh_ < 0.56) range at room temperature must be between M_S_^1^ ~ 825 emu/cm^3^ and M_S_^2^ ~ 1220 emu/cm^3^. The insets of **Fig. 3A** and **Fig. 3B** show~~s~~ **t**he in-plane magnetization M along the [100] direction (hard axis) as a function of the magnetic field H after cooling to nitrogen temperature, heating up to 400K and cooling to room temperature for the A and B samples, respectively.

**Supplementary Figure 4. Temperature dependence of the magnetization of the 45Rh/α_h_**^ʹ^**/55Fe(001** **trilayer obtained by annealing the 45Rh/55Fe bilayer at 350 °C.** The M-T curve was measured with an in-plane magnetic field of 2 kOe. The temperature dependent magnetization curve of the Rh/α_l_^ʹ^/Fe(001) films shows the hysteresis of the AFM ↔ FM transition, which proves the formation of an α_l_^ʹ^ layer between the Rh and Fe (001) films in the 45Rh/55Fe bilayer after annealing at **350 °C.** Similar to the calculations presented in **Supplementary Figure 2,** the contribution ΔM(Fe) = of the unreacted Fe(001) layer towards the magnetization of the Rh/α_h_^ʹ^/Fe(001 trilayer was determined. As follows from Fig. 2c of the manuscript K_4_/K_4_^0^ ~ 0.6, and using expression (3) from Supplementary Note 1 we estimate of the magnetization ΔM(Fe) of the unreacted Fe (001) layer ΔM(Fe) = 0.6 × 875 emu/cm^3^ = 525emu/cm^3^. Subtracting this value from the M-T curve of the Rh/α_l_^ʹ^/Fe (001) trilayer creates the M-T curve of the α_l_^ʹ^ layer with residual magnetization ΔM ~ 35 emu/cm^3^. The relationship (ΔM(α_l_^ʹ^)-ΔM)/ΔM(α_l_^ʹ^) = 0.90 is the degree η of the α_l_^ʹ^→ α^ʺ^ transition which agrees well with the value η = 0.90 obtained by the torque method (**Fig. 2d** of the manuscript). The inset shows the in-plane magnetization M along the [100] direction (hard axis) as a function of the magnetic field H after heating up to 400K and cooling to room temperature.

**Supplementary Figure 5. Temperature dependence of the magnetization of the α_h_^ʹ^ film obtained by annealing the 45Rh/55Fe bilayers at 700** °C**.** The temperature dependence of the magnetization of the 45Rh/55Fe bilayers obtained by annealing at 700 °C measured with an in-plane magnetic field of 1kOe. The obtained α_h_^ʹ^ samples had a high magnetization M_S_ ~ 1270 emu/cm^3^ and did not show an AFM-FM transition. This clearly demonstrates the start of the α_h_^ʹ^ synthesis by the solid-state reaction α_l_^ʹ^ + Fe → (~ 450° C) α_h_^ʹ^, which ends at 500 °C (Fig. 2c of the manuscript).

**Supplementary Figure 6. The formation of a Rh/α_l_^ʹ^(001)** **film in the 68Rh/32Fe(001) bilayer after annealing at 400 °C.**

**a** - TEM image of the cross-section of the **Rh/α_l_^ʹ^ (001)** bilayer. Kirkendall voids are formed on the surface of the MgO(001) as a result of the migration of the Fe atoms into the Rh layer during the solid-phase reaction Fe +Rh → B2-FeRh. **b** - Compositional profiles of Rh and Fe in the Rh/α_l_^ʹ^(001) sample along the scan line shown in the TEM image of the cross-section. Using thickness d_Fe_^0^ =40 nm of the 68Rh/32Fe(001) bilayer and expressions (2) and (6) from **Supplementary Note 1**, we can calculate the thickness of the reacted Fe (001) layer d_Fe_ ~ 40 nm and the α_l_^ʹ^ layer d(α_l_^ʹ^) ~ 100 nm which form in the 68Rh/α_l_^ʹ^/32Fe (001) trilayer after annealing at 400 °C. The calculated value d(α_l_^ʹ^) ~ 100 nm agrees well with the experimental value obtained from the compositional profile presented in (b). **c** - Magnetization vs temperature of the Rh/α_l_^ʹ^ (001) sample after annealing at 400 °C. The M-T curve shows a reversible AFM ↔ FM transition with low residual magnetization and a high degree η = 0.9. This proves that the formed B2-FeRh layer contains the perfect α_l_^ʹ^ phase. The inset shows the in-plane magnetization M along the [100] direction (hard axis) as a function of the magnetic field H after cooling to nitrogen temperature and heating up to room temperature (300 K).

**Supplementary Figure 7. XRD scan of a γ(001) film on MgO(001). a** – XRD scan for the paramagnetic γ(001)layer which was grown epitaxially on a MgO(001) substrate during the solid state reaction between the polycrystalline Pd and epitaxial α_l_^ʹ^ (001) layers above 550 °C. **b** - The φ-2θ asymmetric diffraction images for the γ(0 0 1) film after annealing at 800°C and the MgO substrate. These scans reveal the γ(001)[100]||MgO(001)[100] epitaxial orientation relationship. **c-**Schematic drawing illustrating the cube-on-cube epitaxial relationship of the γ(001) on the MgO(001) substrate.


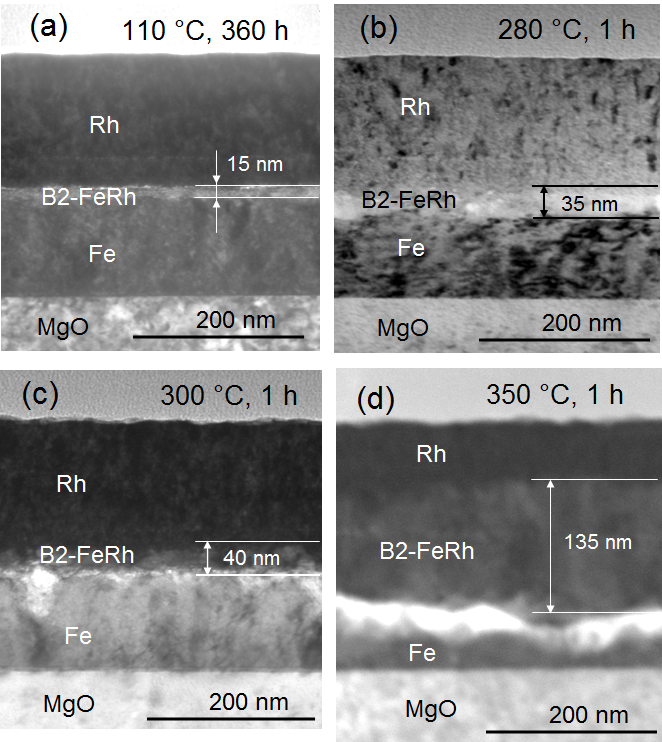


**Supplementary Figure 8. Temperature dependence of the B2-FeRh layer growing on the 52Rh/48Fe interface during annealing from 110ºC to 350 ºC. a -**TEM image of the cross-section of the 52Rh/48Fe bilayer after annealing at 110 ºC for 360h. From Figure 4c of the manuscript, it follows that the thickness of Fe reacted with Rh after aging for 360h at 110 °C is d_Fe_ ~ 8nm. This value, which corresponds to the thickness of the reaction product layer h_FeRh_ = h_nfm-B2_ = 2.15h_Fe_ ~ 17nm, is in good agreement with the experimental value of 15 nm (Supplementary Figure 8a). **b** -280 ºC, 1h, **c** - 300 ºC, 1h and **d** - 350 ºC, 1h showing the evolution of the B2-FeRh layer as function annealing temperature. The dependence K_4_(T_a_)/K_4_^0^ presented in Fig. 1c of the manuscript is equal to ~ 0.53 at a temperature of 350 °C. Using expression (2) and experimental value d_Fe_^0^ = 125 nm the calculated value of d_Fe_ is about 59 nm, which corresponds to d_B2-FeRh_ = 126 nm according to expression (6). The calculated value is somewhat lower than the experimental value 135 nm (Supplementary Figure 8с). After annealing at 350 ºC, it is clearly seen that Kirkendall voids appear on the B2-FeRh/Fe(001) interface. This indicates the predominant migration of Fe atoms through the reaction product B2-FeRh layer into the Rh layer.

**Supplementary Figure 9. change of electrical resistance at the nfm-B2 → α_l_^ʹ^** **transition.** Resistance (R) versus temperature (T) for the 52Rh/48Fe(001) film heated to 500 °C at a 5K/min heating rate and slow cooling to room temperature with a cooling rate of about 2K/min. A sharp change in electrical resistance at 300 °C as the temperature increases indicates a prompt transition from the nonmagnetic nfm-B2 into the ferromagnetic α_l_^ʹ^ phase.

**Supplementary Note 1: Determination of the interlayer thickness d(Fe) and magnetization ΔM(Fe) of the unreacted Fe(001) layer in the Rh/B2-FeRh/Fe(001) trilayer/**

**F**rom **Fig. 1c**, **Fig. 2c** and **Fig. 3c** of the manuscript the K_4_/K_4_^0^ value corresponds to the relative thickness d(Fe)/d_Fe_^0^, where d(Fe) is the unreacted Fe(001) layer thickness and d_Fe_^0^ is the initial Fe(001) layer thickness. Therefore, the thicknesses of the unreacted Fe (001) layer d(Fe) and the reacted Fe (001) layer d_Fe_ = d_Fe_^0^ - d(Fe) are determined by expressions (1) and (2), respectively

d(Fe) = d_Fe_^0^ × K_4_/K_4_^0^ (1)

d_Fe_ = d_Fe_^0^ - d(Fe) = d_Fe_^0^ × (1- K_4_/K_4_^0^ ) (2)

The magnetization of the Rh/B2-FeRh /Fe (001) trilayer at room temperature is the sum ΔM(B2-FeRh) + ΔMFe(001) of the magnetizations of the B2-FeRh and Fe (001) layers. Therefore, the magnetization ΔM(Fe) of the unreacted Fe (001) layer can be found using the expression (3)

ΔM(Fe) = M_S_^0^ × K_4_/K_4_^0^ (3)

Where M_S_^0^ is the magnetization of the original sample ΔM(Fe). ( M_S_^0^ = 825 emu/cm^3^ for 52Rh/48Fe bilayers, M_S_^0^ = 875 emu/cm^3^ for 45Rh/55Fe bilayers and M_S_^0^ = 450 emu/cm^3^ for 68Rh/32Fe bilayers, see Methods).

**Determination of the interlayer thickness d_B2-FeRh_** **in the Rh/B2-FeRh/Fe(001) trilayer if the thickness of the reacted layer d_Fe_ is known**

The equiatomic composition of the B2-FeRh alloys consists of 64.5 wt% Rh. This value is equal to the ratio (4) of the weight of the reacted Rh film layer to the weight of the B2-FeRh film which is formed in the reaction products.

(ρ_Rh_·d_Rh_·S)/(ρ_Rh_·d_Rh_·S + ρ_Fe_·d_Fe_·S) = 0.645 (4),

where S is the film area and ρ_Rh_, ρ_Fe_ and d_Rh_, d_Fe_ are the densities and thicknesses of the reacted Rh and Fe layers, respectively. From expression (4) follows (5), (6):

d_Rh_ = (0.645ρ_Fe_/0.355ρ_Rh_)d_Fe_ = 1.15d_Fe_ (5)

The thickness d_B2-FeRh_ is the sum of the thicknesses d_Rh_ and d_Fe_ of the reacted Fe and Rh layers (6)

d_B2-FeRh_ = d_Rh_ + d_Fe_ = 2.15d_Fe_ (6)

**Supplementary Note 2: Determination of the thickness d**(α_l_^ʹ^**) in the 52Rh/**(**nfm-B2+α_l_^ʹ^)/48Fe(001) trilayer annealed at 300 °C.**

Fig. 5b of the manuscript shows the M-T curve, which is an attribute of the α_l_^ʹ^ phase formation. The change in magnetization during the heating and cooling ΔM (α_l_^ʹ^) is about 60 emu/cm^3^. For a perfect α_l_^ʹ^ phase, the magnetization is M(α_l_^ʹ^) = 825 emu/cm^3^ and the ratio ΔM(α_l_^ʹ^)/M (α_l_^ʹ^) is approximately equal to the ratio of the thicknesses d(α_l_^ʹ^)/d(B2-FeRh), where d(B2-FeRh) is the thickness of the fully reacted sample 50Rh/50Fe. This thickness is approximately equal to the thickness of the original 48Rh/52Fe(001) sample (7).

ΔM(α_l_^ʹ^)/M(α_l_^ʹ^) ~ d(α_l_^ʹ^)/d(52Rh/48Fe(001) (7)

Using equation (7) and the experimental value h(48Rh/52Fe(001) =270 nm the calculated value of d(α_l_^ʹ^) is about 20 nm. From Supplementary Figure 8c it follows that the thickness h(B2-FeRh) ~ 35 nm, which is the sum of the h(α_l_^ʹ^) and h(nfm-B2) layers. Thus, after annealing at 300 °C, the reacted B2-FeRh layer contains a two-phase mixture of ~ 40% non-magnetic nfm-B2 and ~ 60% ferromagnetic α_l_^ʹ^.

**Supplementary Note 3: The possibility of the fabrication of the B2-FeRh compound by self-propagating synthesis.**

It is well known that the B2-NiTi [3], B2-AlNi [4,5] and B2-AuCd [6] compounds not only possess low-temperature martensitic transformations, but can also be synthesized by self-propagating high-temperature synthesis (SHS) in powders and multilayer systems. These studies are described in numerous papers and continue to be investigated intensively (see, e.g., Refs. [7- 21]). The key parameter for a reaction to be self-sustaining is the temperature of adiabatic combustion T_ad_ which for many SHS systems follows the empirical rule T_ad_ > 1800 K [10]. The expression T_ad_ = T_0_ + ΔH^0^/C_p_ is often used for rough estimates of T_ad_, where T_0_, ΔH^0^, and C_p_ are the initial temperature, enthalpy of the reaction and molar specific capacity, respectively. For reactions between Ni and Ti and Ni and Al the enthalpy of the reaction ΔH^0^ is equal to the standard enthalpies of formation ΔH_f_º(B2-NiTi) = - 31.1 kJ/mol [19] and ΔH_f_º(B2-NiAl) = - 62 ± 2 kJ/mol [20], which correspond to the calculated temperatures T_ad_(B2-NiTi) = 1540K, T_ad_(B2-NiAl) = 2780K, for the B2-NiTi and B2-NiAl compounds, respectively. The real values of the adiabatic temperatures T_ad_(B2-NiTi) = 1552 K [10] and T_ad_(B2-NiAl) = 1911 K [10] agree well with the calculated value for B2-NiTi but are much smaller for the B2-NiAl compound. For the combustion synthesis of B2-TiNi the relatively low adiabatic temperature T_ad_(B2-NiTi) must be increased by increasing T_0,_ which means the reactants must be heated above 150 ºC [7]. The experimental value of the enthalpy of formation ΔH_f_º(B2-FeRh) of the B2-FeRh compound is unknown, however, the magnitude of ΔH_f_º(B2-FeRh) = - 26.6 kJ/mol was calculated by Eleno et al. using the Cluster Variation Method [21]. This value at the initial temperature T_0_ =273 K corresponds to the adiabatic temperature T_ad_(B2-FeRh) = 1340K. The close values of the initiation and adiabatic temperatures and formation enthalpies for the B2-FeRh and B2-NiTi phases clearly suggests the possibility of the fabrication of the B2-FeRh compound by SHS Rh using preheating in the reactive systems similar to the combustion synthesis of B2-NiTi [7].

**Supplementary References:**

1. Yamada, S. et al., Exchange coupling in metallic multilayers with a top FeRh layer. *AIP Advances* **6,** 056115, (2016). 2. Suzuki, I., Hamasaki, Y., Itoh, M. & Taniyama, T. Controllable exchange bias in Fe/metamagnetic FeRh bilayers. *Appl. Phys. Lett.* **105,** 72401, (2014). 3. [Otsuka](https://www.sciencedirect.com/science/article/pii/S0079642504000647?via%3Dihub#!), K., & [Ren](https://www.sciencedirect.com/science/article/pii/S0079642504000647?via%3Dihub#!), X. Physical metallurgy of Ti–Ni-based shape memory alloys. [*Prog. Mater. Sci.*](https://www.sciencedirect.com/science/journal/00796425) **50,** 511-678, (2005)**.** 4.  [Potapov](https://www.sciencedirect.com/science/article/pii/1359646296001364#!), P. L., [Poliakova](https://www.sciencedirect.com/science/article/pii/1359646296001364#!), N. A. & [Udovenko](https://www.sciencedirect.com/science/article/pii/1359646296001364#!), V. A. The shape memory behavior in 63.8Ni-Al alloy. *Scripta Mater.* **35,** 423-427, (1996). 5. Zhang, H., Salje, E. K. H., Schryvers, D. & Bartova, B. The martensitic phase transition in Ni–Al: experimental observation of excess entropy and heterogeneous spontaneous strain. *J. Phys.: Condens. Matter.* **20,** (2008) 055220, (2008). 6. Cahn, R. W. Metallic rubber bounces back. *Nature* **374,** 120-121, (1995). 7. Li, B. Y., Rong, L. J., Li, Y. Y. & Gjunter, V. E. Synthesis of porous Ni–Ti shape-memory alloys by self-propagating high-temperature synthesis: reaction mechanism and anisotropy in pore structure. *Acta Mater.* **48**, 3895–3904, (2000). 8. [Singh, S.](https://www.scopus.com/authid/detail.uri?origin=resultslist&authorId=57206711836&zone=), [Swain, M.](https://www.scopus.com/authid/detail.uri?origin=resultslist&authorId=55667901900&zone=) & [Basu, S.](https://www.scopus.com/authid/detail.uri?origin=resultslist&authorId=7403656041&zone=) [Kinetics of interface alloy phase formation at nanometer length scale in ultra-thin films: X-ray and polarized neutron reflectometry](https://www.scopus.com/record/display.uri?eid=2-s2.0-85044569322&origin=resultslist&sort=plf-f&cite=2-s2.0-0034271667&src=s&imp=t&sid=2714b0d1c8677ec004404c199ee18bcb&sot=cite&sdt=a&sl=0&relpos=5&citeCnt=2&searchTerm=). [*Prog. Mater. Sci*.](https://www.scopus.com/sourceid/17899?origin=resultslist) **96,** 1-50, (2018). 9. Hua, L., Xue, Y. & Shi, F. Intermetallic formation and mechanical properties of Ni-Ti diffusion couples. [*Materials and Design*](https://www.scopus.com/sourceid/17797?origin=recordpage) **130,** 175-182, (2017)**.** 10. Munir, Z. A. & Anselmi-Tamburini, U. [Self-propagating exothermic reactions: the synthesis of high-temperature materials by combustion](javascript:void(0)), Mater. *Sci. Rep.* **3,** 277-365, (1989). 11. Myagkov, V. G., Bykova, L.E., Zharkov, S. M. & Bondarenko, G. N. Formation of NiAl shape memory alloy thin films by solid-state reaction. *Solid State Phenomena* **138,** 377-384, (2008). 12. Myagkov, V. G., Mikhlin, Yu. L., Bykova, L. E., Bondarenko, G. V. & Bondarenko, G. N. Long-Range Nature of Chemical Interaction in Solid-Phase Reactions: Formation of Martensite Phases of an Au–Cd Alloy in Cd/Fe/Au Film Systems. *Doklady Physical Chemistry* **431,** 52-56, (2010). 13. Myagkov, V. G., Bykova, L. E. & Bondarenko, G. N. Solid-State Synthesis and Martensitic Transformations in Thin Films. [*Doklady Physics*](http://www.maik.ru/cgi-perl/journal.pl?lang=rus&name=danphys) **48**, 30 – 33, (2003). 14. [Fritz](https://aip.scitation.org/author/Fritz%2C+Gregory+M), G. M., [Spey Jr.](https://aip.scitation.org/author/Spey%2C+Stephen+J+Jr), S. J., [Grapes](https://aip.scitation.org/author/Grapes%2C+Michael+D), M. D. & [Weihs](https://aip.scitation.org/author/Weihs%2C+Timothy+P), T. P. Thresholds for igniting exothermic reactions in Al/Ni multilayers using pulses of electrical, mechanical, and thermal energy. *J. Appl. Phys.***113** 01490, (2013). 15. Weihs, T.P. Fabrication and characterization of reactive multilayer films and foils, in: K. Barmak, K. Coffey (Eds.), Metallic Films for Electronic, Optical and Magnetic Applications: Structure, Processing and Properties, (2014) 160-243. 16. Myagkov, V. G., & Bykova, L. E. Solid-Phase Synthesis and Martensitic Transformations in Al/Ni Thin Films. *Doklady Physics* **49**, 289–291, (2004). 17. Myagkov, V. G. Ultrafast Solid-State Reactions and Martensitic Transformations in Thin Films. *Doklady Physics* **48**, 495–498, (2003). 18. Myagkov, V. G., Bykova, L. E. Li, L. A., Turpanov, I. A., & Bondarenko, G. N. Solid-Phase Reactions, Self-Propagating High-Temperature Synthesis, and Martensitic Transformations in Thin Films, Doklady Physics **47**, 95–98, (2002). 19. Moser, Z., Gasior, W., Rzyman, K. & Debski, A. Calorimetric studies of the enthalpies of formation of NiTi_2_, NiTi and Ni_3_Ti,  *Archives of Metallurgy and Materials* **51**, 606-608, (2006). 20. Hu, R. & Nash, P., The enthalpy of formation of NiAl. *J. of Mater. Sci.,* **40** 1067 – 1069, (2005). 21. [Ohnuma](https://www.jstage.jst.go.jp/search/global/_search/-char/en?item=8&word=Ikuo+Ohnuma), I., [Gendo](https://www.jstage.jst.go.jp/search/global/_search/-char/en?item=8&word=Toshiyuki+Gendo), T., [Kainuma](https://www.jstage.jst.go.jp/search/global/_search/-char/en?item=8&word=Ryosuke+Kainuma), R. [Inden](https://www.jstage.jst.go.jp/search/global/_search/-char/en?item=8&word=Gerhard+Inden), G. & [Ishida](https://www.jstage.jst.go.jp/search/global/_search/-char/en?item=8&word=Kiyohito+Ishida), K. Phase equilibria and thermodynamic evaluation approximating short-range ordering energy in the Fe–Rh Binary system. [*ISIJ International*](https://www.jstage.jst.go.jp/browse/isijinternational/-char/en) **49** 1212-1219, (2009).
